# Oral Secretions

**Published:** 1878-06

**Authors:** G. S. Shattuck


### ORAL SECRETIONS.

BY G. S. SHATTUCK, D. D. S.

Mr. President—Gentlemen:—I desire to present a few thoughts upon the chemistry of the oral secretions.

Of all the subjects which the dentist has to consider in the rounds of his daily practice, the oral secretions stand high in the list. But a majority of them give it but little thought. They do not seem to realize the important part this fluid plays in "taking down the lime salts," of which the teeth are chiefly composed, or protecting the teeth while nature is repairing an injury.

There seems to be a tendency on the part of dental practitioners to treat and repair the effects, and pay little or no attention to the causes. Many of them never give the chemistry of these or any other secretions a moment's thought; while a few of the more liberally educated among us with litmus, microscope and test tube are searching after the active elements which make such inroads upon the teeth.

In speaking of the oral secretions, we mean those secreted by the glands whose ducts open into the oral cavity with which you are all familiar. The saliva from the parotid gland is pure and clear. The secretion from the sublingual is the same as that from the parotid with the addition of sulpho-cyanide of potash.

The secretion from the submaxillary is more viscid and ropy. The specific gravity varies from one thousand and two to one thousand and four, taking water as the standard one thousand. We do not find the saliva as pure as when secreted by the several glands. The organic decompositions are so great, and so varied, that to take a sample of saliva and give different portions to different chemists, no two of them will send in the same results.

Physiology teaches us that, in order to properly protect the teeth against acid taken by food or arising from any cause, the saliva should have an alkaline reaction. But we do not always find it so. We find it acid in a majority of cases. In one hundred cases examined by the writer it was found acid in seventy-five, neutral in three and alkaline in twenty-two.

By taking the results of a great many analyses, I find the following substances have been detected in the saliva:

Salts of lime and soda generally as phosphates, and to these the alkalinity is undoubtedly due. Calculi of salts of lime are not very unfrequently found. The so-called tartar which is deposited upon the teeth, is composed of phosphate of lime and some organic matter.

The acids are hydrochloric, nitric, sulphuric, carbonic, acetic, oxalic, uric, lactic, etc.

The chemical test for hydrochloric acid is to add to a small portion of saliva in a test tube, a few drops of a solution of nitrate of silver, and if any HCl be present, a white precipitate will be formed, insoluble in acid, soluble in ammonia.

Sulphuric acid has been found once by Prof. Vaughn, I believe, but we have seen results in many cases which lead us to believe it must have been present as in "black decay," and it is not at all improbable that it is present. In all albuminoids we find sulphur, and by the action of nitric acid upon this, it would be changed into sulphuric acid. Chlorine and water would do the same thing. A great many other oxidizing agents would also have the same result. The chemical test for sulphates or sulphuric acid, is to add a solution of barium chloride, and if any sulphuric acid is present, a white precipitate is formed insoluble in acid or alkalies. A great deal of the food contains nitrogenous material, and nitrogen is liberated from this in one of many ways as an oxide or as ammonia ($NH_3$), and in either case would be changed into nitric acid by further oxidation. The chemical test for nitric acid is, add to a small portion of sulphuric acid in a test tube some ferrous sulphate, then drop in some saliva, and if nitric acid is present a brown ring will be formed.

Acetic acid is taken with the food. The chemical test for phosphates is to add a solution of Uric acetate and Sodic acetate, and a white precipitate will be formed.

Uric acid is sometimes found in the saliva; its presence is probably due to the failure of the kidneys to perform their work. The Uric acid is left in blood, and may be in the saliva from this cause. Long retention of urine may also have some effect. The test for uric acid is to evaporate a small portion of Ammonic hydrate, and if any uric acid is present, you will get a crimson color.

Urea may be present, and may be detected by the microscope after adding nitric acid to a small portion of saliva on a glass slide, crystals of nitrate of urea will be formed. They are rhombic plates, soluble in water.

Urates may also be present, and may be decomposed by nitric acid, and the test for uric acid applied.

Lactic acid is formed by the decomposition of lactate of soda. This is not found very often.

Carbonic acid is found. The chemical test is by adding acetic acid and examine under the microscope for effervescence of carbonic anhydride.

Oxalic acid is found also. It is one of the fruit acids. The chemical test is, add solution of calcium and oxalate of calcium—crystals can be detected under the microscope. They are octahedral.

Sulpho-cyanide of potash is secreted by sublingual gland. It may be tested for by adding some ferric chloride and a blood red color will be the result. This color is destroyed by the addition of a solution of corrosive sublimate.

Albumen is found in the saliva. The test is heat and it will be coagulated and this will not be soluble in acids.

Mucine is found in saliva, and to this the saliva owes its viscidity.

Ptyaline is found, and may be obtained for practical examination, by digesting the parotid gland of an ox or sheep in alcohol for twenty-four hours, then strain and place in glycerine and strain again. The ptyaline being soluble in glycerine, would be left from any coloring matter which the alcohol might have dissolved, and will appear in white flakes.

Pus and blood may also be found, and can be seen under the microscope. There will be an excess of albumen if either blood or pus is present.

Cholestrine may be present. It comes from the nervous system. It is soft like albumen, soluble in hot alcohol, deposited when cold in flat plates. The microscopical test is the best one. When pure if sulphuric acid is added, you will get a play of colors.

Milky saliva is sometimes found in females during lactation. Contains oil globules which may be seen under the microscope.

The sugar found in saliva may be of two kinds, grape and lactose. Grape sugar may be in saliva because there is a superabundance, or, because it is not assimilated. Lactose very rarely occurs in saliva. Only one case recorded, and that one doubtful. Sometimes medicinal substances taken internally are found in the saliva, as iodine, bromine, mercuric chloride, etc.

According to Berzelius the teeth consist of the following substances:

Phosphate of lime, carbonate of lime, fluoride of lime, magnesic phosphate, soda chloride, gelatine and water. All of which salts, with the exception of fluoride of lime, are soluble in the acids found in saliva. The fluoride of lime is, however, sparingly soluble in cold nitric or sulphuric acids. So we can readily see the effects of the oral secretions upon the teeth when they remain in this abnormal condition for any length of time.

